# Male circumcision for HIV prevention - a cross-sectional study on awareness among young people and adults in rural Uganda

**DOI:** 10.1186/1471-2458-10-209

**Published:** 2010-04-26

**Authors:** Andrea Wilcken, Flavia Miiro-Nakayima, Ramadhan NB Hizaamu, Thomas Keil, Dorothy Balaba-Byansi

**Affiliations:** 1 Traditional and Modern Health Practitioners Together against AIDS (THETA), Kampala, Uganda; 2 Institute for Social Medicine, Epidemiology and Health Economics, Charité University Medical Centre, Berlin Germany

## Abstract

**Background:**

Medical male circumcision is now part of a comprehensive approach to HIV prevention. It has been shown that awareness of the protective effect of male circumcision leads to high acceptability towards the introduction of medical male circumcision services within countries. The objective of this survey was to identify factors determining awareness of male circumcision for HIV prevention.

**Methods:**

We interviewed 452 participants (267 adults >24 years of age; 185 youths 14-24 years) living in three rural Ugandan districts in 2008. Using a standardized questionnaire, we assessed socio-demographic parameters, awareness of MC for HIV prevention, general beliefs/attitudes regarding MC and MC status. Determinants for awareness of MC for HIV prevention were examined with multiple logistic regression models.

**Results:**

Out of all adults, 52.1% were male (mean ± SD age 39.8 ± 11 years), of whom 39.1% reported to be circumcised. Out of all youths, 58.4% were male (18.4 ± 2.5), 35.0% circumcised.

Adults were more aware of MC for HIV prevention than youths (87.1% vs. 76.5%; p = 0.004). In adults, awareness was increased with higher educational level compared to no school: primary school (adjusted OR 9.32; 95%CI 1.80-48.11), secondary (5.04; 1.01-25.25), tertiary (9.91; 0.76-129.18), university education (8.03; 0.59-109.95). Younger age and male sex were further significant determinants of increased awareness, but not marital status, religion, district, ethnicity, employment status, and circumcision status. In youths, we found a borderline statistically significant decrease of awareness of MC for HIV prevention with higher educational level, but not with any other socio-demographic factors.

**Conclusions:**

Particularly Ugandans with low education, youths, and women, playing an important role in decision-making of MC for their partners and sons, should be increasingly targeted by information campaigns about positive health effects of MC.

## Background

With 2.7 million new HIV infections and two million HIV-related deaths globally in 2007 [1], the need to strengthen existing preventive strategies against HIV/AIDS is at hand. Male circumcision (MC), to some religious and ethnic groups a centuries-old practice, has been shown to reduce the risk of female to male transmission of HIV by at least 50%[2-4] The protective effect of MC is expected to have its greatest impact in countries where HIV prevalence is high and MC is little practised, most of them being countries of Sub-Saharan Africa[5] Large-scale implementation of MC could potentially lead to a reduction of HIV prevalence up to 67% considering that the main mode of HIV transmission is through heterosexual contact[6] In Uganda, the prevalence of HIV above the age of 15 years is 5.4%[1] MC is not a common practice; only 24% of males are circumcised. Approximately 12% of Ugandans are Muslims, conforming to the religious practice of being circumcised[7,8] It is estimated that 14% of Ugandans are circumcised for non-religious reasons[9] MC is more common among urban than rural men, it is thus more common in Kampala than elsewhere in the country except for the Eastern and in the mountains of the Western region where MC is carried out as a rite of passage among the Bagisu, Sabiny and Bakonzo and 80% of males are reportedly circumcised[8] In addition, a few men are circumcised for medical reasons and, following public awareness of the results of research on medical MC for HIV prevention in Rakai, men started seeking medical MC services to reduce their risk of contracting HIV/AIDS. Studies have shown that acceptability for the introduction of MC into traditionally non-circumcising communities in Uganda, Kenya, Tanzania, Malawi, Zambia, Zimbabwe, Botswana, Swaziland and South Africa is high[10] Protection from sexually transmitted diseases (STDs) and HIV was a condition for acceptability in some of the countries [11-14] and willingness to become circumcised and, for male and female study participants, the willingness to consider MC for their sons, has been shown to increase significantly after information sessions[11,15] Exposure to different health beliefs and practices through mixing of ethnic groups in secondary schools or urban areas could also contribute to increased awareness of MC and adoption of the practice[16].

The aim of our study was to identify socio-demographic factors that are associated with the awareness of MC for HIV prevention among youth and adult residents of rural communities in Uganda. Furthermore, we assessed male participants' circumcision status, circumcision practices and general knowledge on MC, attitudes in favour and against MC among female and male participants of the study.

## Methods

### Study design and setting

This cross-sectional study was part of a larger survey that was carried out in rural areas of three districts (Mpigi, Kayunga, Kaliro) in Central and Eastern Uganda in June 2008. The larger survey was meant to support the development of age-specific preventive strategies against HIV for young people and adults.

### Sampling method and study population

The districts where the survey was conducted were purposely selected based on data from the HIV/AIDS Sero-Behavioural Survey 2004-05 showing a relatively high prevalence of HIV in Eastern and Central Uganda[8] Each district consists of 3 to 4 counties with a differing number of sub-counties. Two sub-counties within one county of each of the 3 districts were selected after consulting the local health administration and avoiding overlaps with HIV prevention programmes by other service providers. In each sub-county, three out of 6 to 8 parishes were randomly chosen. The local chair persons of the sub-counties were asked to consecutively select every fifth household registered. The first person from each of the two age strata encountered in each household was interviewed, i.e. youths between 14 and 24 years of age and adults above the age of 24 years. For the adults, three subgroups were recruited: Parents, teachers, "Sengas" and "Kojjas". In Luganda and Lusoga, "Senga" literally means "paternal auntie", "Kojja" literally means "maternal uncle". Traditionally, Sengas and Kojjas offer counselling and guidance on relationships, marriage strengthening, sexuality, reproductive health practices for couples and young people in the communities. For Sengas and Kojjas, snowball sampling was used. The sample size for the general survey was calculated using the simplified formula for proportions n = N/1+N*e^2 ^(assuming a 95% confidence level and a proportion (e) of 50%) [17]. With a level of precision of e = 0.05 and a population size of N = 9000, this yielded a target sample size of n = 383). We added 15% to this sample size to compensate for persons not available and increased the sample size by another 30% to compensate for non-response. This yielded a target sample size of 555 persons. Approval for the study was obtained from the health authorities of the respective districts, i.e. the District Director of Health Services, the Chief Administrative Officer and the Sub-County Chief. Ethical approval for data analysis and publication was obtained by the review board of Charité University Medical Center Berlin, Germany.

### Interviews and data management

We developed a standardized questionnaire that was translated into Luganda, back-translated to ensure accuracy of the Lugandan version and pretested among 20 youths and adults before data collection started. Research assistants received a two-day training workshop before they conducted face-to-face interviews in the local language. Data was entered into Epi Info™ Version 6.04 (Center for Disease Control and Prevention, Atlanta, Georgia, USA; http://www.cdc.gov/epiinfo/).

### Assessment of outcome variables

The primary outcome was assessed by the closed question "Have you ever heard that male circumcision reduced the risk of contracting HIV?" (Yes/No). In addition, we used open-ended questions to assess general knowledge on MC ("What do you know about circumcision?") as well as attitudes in favour and against MC. The open-ended questions were asked before the closed questions. Circumcision status of male participants, sons of participants and male circumcision practices were assessed by closed questions.

### Assessment of socio-demographic variables

Based on standardized questions, we assessed age, sex, marital status, religious affiliation, tribal identity, educational level, employment status, district of current residence, and social role/function in the group of adults (parents, Sengas/Kojjas or teachers) to examine a possible association of these variables with the primary outcome, e.g. awareness of MC for HIV prevention.

### Statistical methods

Following the design of the larger survey, we performed all analyses separately for the two target groups (youths aged 14-24 years and adults above the age of 24 years). Comparing basic characteristics between youths and adults we tested differences by chi-squared test (for categorical variables) and t-test or, in case of non-parametric distribution, Mann-Whitney-U-test (for continuous variables). Two-sided *p *values of less than 0.05 were considered statistically significant. Stratified for youths and adults, we calculated odds ratios (OR) with 95% confidence intervals (CI) as a measure of uncertainty to estimate the associations between the knowledge that MC reduces the risk of contracting HIV and different socio-demographic parameters. In multiple logistic regression analyses, we calculated adjusted ORs and 95%CIs for sex, age (in years), district (Kaliro, Kayunga, Mpigi), religion (Catholic, Protestant, Muslim), tribe (Musoga, Muganda, Mulamoga, Munyara, others), educational level (no school, primary, secondary, tertiary school, university), and marital status (never married, married/cohabiting, separated/widowed). In addition, for adults, work status (no work, salaried, self-employed, casual/manual work), salary (as a continuous variable) and adult subgroup (parents, Sengas/Kojjas, teachers) were included in the model. Regardless of statistical significance in univariate analyses, all variables were included en bloc to evaluate a broad range of determinants for awareness of MC for HIV. All calculations were performed using the statistical software package SPSS 16.0 (SPSS Inc., Chicago, Illinois, USA).

## Results

### Basic characteristics

We approached 560 residents, of whom 452 participated in the interviews. Thus, the response rate was 81%. 185 were "youth" between 14 and 24 years of age, 267 were adults above 24 years of age, hereof 41.6% parents (median age 38 years), 34.8% Sengas/Kojjas (45 years) and 23.6% teachers (30 years). Age differences between these three adult sub-groups were significant (p < 0.001).

**Figure 1:**
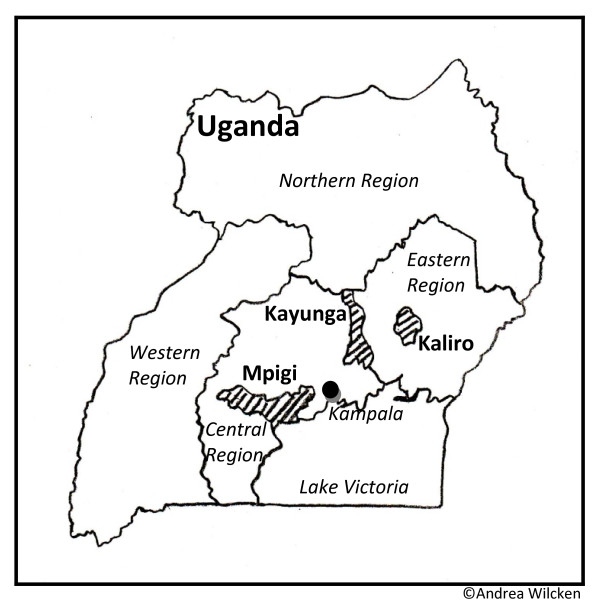
**Self-reported male circumcision rates and ages at male circumcision per district**. **Kaliro: **males circumcised: 18.4%; median age at male circumcision (IQR): 15 yrs (5.25-21.25 yrs). **Kayunga: **males circumcised: 33.3%, median age at male circumcision (IQR): 16 yrs (5-19 yrs). **Mpigi: **males circumcised: 48.3%, median age at male circumcision (IQR): 4 yrs (0-8 yrs)

More than half of all participants had completed secondary education; two thirds were of Christian faith, the remaining mostly Muslims. Participants were from 26 different tribes, mainly Baganda and Basoga, which are the indigenous tribes in the study region (Table 1). More than one third of all male participants (37.3%) reported to be circumcised. The median age at circumcision was not significantly different between youths and adults (7.5 vs. 12 years, p = 0.393). Circumcision rates differed significantly between the three districts (p < 0.001) and age at MC was significantly lower in Mpigi as compared to Kaliro and Kayunga (Figure 1). It was significantly higher among participants that were circumcised as a rite of passage into manhood (16 vs. 6.5 years, p = 0.021). The majority of respondents (65.0%) were circumcised by a traditional provider, adults more often than youth (73.5% vs. 53.8%, p = 0.113). The performance of MC as a rite of passage into manhood was mentioned by 19.6%. More than half of participants' sons (N = 102/196) were reportedly circumcised, 54% as infants and 90.8% until 9 years of age. Circumcision had been carried out by a traditional provider in 58.0%.

**Table 1:** Basic characteristics of the study participants (N = 452)

	Youth (N = 185)		Adults (N = 267)		p-Value
	**(N)**	**(%)**	**(N)**	**(%)**	

**Male Sex**	108	58.4	139	52.1	p = 0.185

**Age (mean +/- SD; median)**	185	18.4 +/- 2.5; 18.0	267	39.8 +/- 11.0; 38.0	p < 0.001

**Marital status**					p < 0.001

- **Married**	34	20.7	24	87.1	

- **Separated/divorced/widowed**	6	3.7	229	9.1	

- **Never married**	124	75.6	10	3.8	

**Religion**					p = 0.020

- **Catholic**	36	19.5	31	11.7	

- **Protestant**	89	48.1	144	54.3	

- **Muslim**	57	30.8	90	34.0	

- **Other**	3	1.6	0	0.0	

**Tribe**					p = 0.060

- **(Mu)Soga**	57	31.0	94	36.0	

- **(Mu)Ganda**	72	39.1	111	42.5	

- **Mulamoga**	13	7.1	6	2.3	

- **Munyara**	10	5.4	7	2.7	

- **Other**	32	17.4	43	16.5	

**Education completed**					p < 0.001

- **No school**	1	0.6	16	6.0	

- **Primary**	76	42.9	103	38.9	

- **Secondary**	95	53.7	96	36.2	

- **Tertiary**	3	1.7	30	11.3	

- **University**	2	1.1	20	7.5	

**Work**					p < 0.001

- **Self-employed (agriculture, selling, service)**	33	21.3	136	52.9	

- **Salaried**	19	12.3	83	32.3	

- **Manual/casual Work**	7	4.5	6	2.3	

- **No work/unemployed**	69	61.9	32	12.5	


### Awareness of male circumcision for HIV prevention

Adults were aware of MC as a means of HIV prevention significantly more often than young people (87.1% vs. 76.5%; p = 0.004) with no differences among parents, Sengas/Kojjas and teachers (p = 0.930). Most frequently, the media were mentioned as source of information on that matter (56.5% of youth vs. 62.2% of adults; p = 0.316), followed by information through friends and family (19.7% of youth vs. 20.4% of adults; p = 0.875). Young people reported less often than adults that they had received information on of MC for HIV prevention by health workers (4.9% vs. 10.9%; p = 0.062); instead, teachers and counsellors were the third important source of information for the youth (11.5% vs. 3.0%; p = 0.002). People who are circumcised were also reported as providers of information, significantly more often by male participants (8.2% vs. 2.9%; p = 0.043).

### Logistic regression analysis

#### Adults

In univariate logistic regression analysis, the likelihood for awareness of MC for HIV prevention was most pronounced for higher educational level. The effect estimates remained statistically significant for primary and secondary education and borderline significant for tertiary education after adjusting for district, sex, age, marital status, religion, ethnic group, adult subgroups, employment status and income. No statistically significant effects were found for age, marital status, religion, district, ethnic group, employment status in univariate analysis. In the subgroup of men, circumcision status was not associated with awareness of MC for HIV prevention either (Table 2). However, the effect of slightly decreased awareness with older age became statistically significant after adjusting for district, sex, educational level, marital status, religion, ethnic group, adult subgroups, employment status and income. Likewise, the effect estimate for awareness of MC for HIV prevention for male sex increased further and was borderline statistically significant after adjusting. Participants who are or were married were 5-6 times more likely to be aware of MC as a means of HIV prevention compared to those who never married (adjusted analyses, Table 2).

**Table 2:** The likelihood for awareness of male circumcision for HIV prevention for *adults *(N = 219) in univariate and multiple logistic regression analyses*; statistically significant results bold

	Crude OR	95% CI	p-value	Adjusted OR	95% CI	p-value
**Male sex **(N = 137/263)	1.44	0.70-2.98	0.320	2.78	0.91-8.52	0.073

**Age **(N = 263)	0.98	0.95-1.01	0.207	**0.95**	**0.90-0.99**	**0.028**

**Adult subgroup **(N = 263)						

- **Parents **(N = 109)	1	-	-	1	-	-

- **Sengas/Kojjas **(N = 92)	1.18	0.51-2.70	0.704	1.24	0.37-4.20	0.730

- **Teachers **(N = 62)	1.08	0.43-2.71	0.874	0.33	0.03-3.63	0.362

**Educational level **(N = 261)						

- **no school **(N = 15)	1	-	-	1	-	-

- **primary **(N = 101)	**5.46**	**1.63-18.25**	**0.006**	**9.32**	**1.80-48.11**	**0.008**

- **secondary **(N = 95)	**4.21**	**1.28-13.78**	**0.018**	**5.04**	**1.01-25.25**	**0.049**

- **tertiary **(N = 30)	**9.33**	**1.59-54.67**	**0.013**	9.91	0.76-129.18	0.080

- **university **(N = 20)	**6.00**	**1.003-35.91**	**0.050**	8.03	0.59-109.95	0.119

**Religion **(N = 261)						

- **Catholic **(N = 31)	1	-	-	1	-	-

- **Protestant **(N = 141)	0.85	0.27-2.67	0.776	0.33	0.05-2.03	0.229

- **Muslim **(N = 89)	1.32	0.38-4.62	0.668	0.62	0.09-4.44	0.633

**Tribe **(N = 257)						

- **Musoga **(N = 92)	1	-	-	1	-	-

- **Muganda **(N = 110)	1.44	0.66-3.14	0.355	1.13	0.21-6.19	0.887

- **Mulamoga **(N = 5)	not estimable			not estimable		

- **Munyara **(N = 7)	1.26	0.14-11.23	0.834	1.86	0.08-43.66	0.700

- **Other **(N = 43)	4.32	0.95-19.70	0.059	3.47	0.47-25.64	0.222

**District **(N = 263)						

- **Mpigi **(N = 87)	1	-	-	1	-	-

- **Kaliro **(N = 90)	0.63	0.26-1.53	0.306	0.83	0.10-7.03	0.866

- **Kayunga **(N = 86)	0.79	0.31-2.01	0.616	1.59	0.33-7.62	0.566

**Work **(N = 253)						

- **no work **(N = 32)	1	-	-	1	-	-

- **salaried **(N = 82)	2.14	0.68-6.73	0.196	2.34	0.30-18.57	0.421

- **self-employed **(N = 133)	1.47	0.53-4.08	0.454	1.47	0.40-5.39	0.560

- **casual/manual work**	not estimable (N = 6)			not estimable		

**Salary **(continuous variable; N = 236)	1.00	1.00-1.00	0.724	1.00	1.00-1.00	0.929

**Marital status **(N = 259)						

- **never married **(N = 10)	1	-	-	1	-	-

- **married/cohabiting **(N = 226)	1.77	0.36-8.75	0.485	5.28	0.55-50.80	0.150

- **separated/widowed **(N = 23)	1.19	0.18-7.84	0.858	6.42	0.38-108.05	0.197

**Circumcised males **(N = 52/132)	1.19	0.38-3.78	0.766	-	-	-

**Age at circumcision **(N = 36/52)	1.24	0.89-1.73	0.197	-	-	-

* Variables included in the adjusted model were sex, age (as a continuous variable), adult subgroup, educational level, religion, tribe, district, employment status, salary, marital status

#### Youth

In univariate logistic regression analysis, awareness of MC for HIV prevention was significantly lower in youths with higher educational level, i.e. secondary education (N = 100) as compared to primary education (N = 74) (OR = 0.35, 95% CI 0.16-0.77, p = 0.009). Odds ratios for the five categories of education level in the adults' subgroup were not estimable for the youth ("no school" N = 1; "tertiary" N = 3; "university" N = 2). The likelihood for awareness of MC for HIV prevention was increased for male sex (N = 107, OR = 1.15, 95% CI 0.58-2.30, p = 0.686), Muslim religion (N = 56) as compared to Catholic religion (N = 36) (OR = 1.31, 95% CI 0.46-3.73, p = 0.607) and being married/cohabiting (N = 34) as compared to never married (N = 122, OR = 2.23, 95% CI 0.73-6.99, p = 0.162). However, these effect estimates were not statistically significant. Age, employment status, district, ethnic group, and circumcision status (in the subgroup of males) were not significantly associated with the awareness on MC for HIV prevention. After adjusting for sex, age, marital status, religion, district and ethnic group the effect for educational level was borderline statistically significant (OR = 0.41, 95% CI 0.15-1.10, p = 0.076). None of the other variables included in the adjusted model (sex, age, marital status, religion, district and ethnic group) showed statistically significant effects. Age and educational level were not correlated; therefore, both variables could remain in the adjusted models.

### General beliefs and reasons for and against male circumcision

Only 1.6% of the participants had never heard about MC. Open-ended questions on general knowledge and beliefs were answered by 82% (multiple answers were possible). In contrast to the results of the specific question on MC for HIV prevention, only one third (38.2%) mentioned MC as a protective measure against HIV in the open-ended question. One third (33.9%) associated MC with religious or cultural practice. Hygiene and protection against STDs were associated with MC by 15.4% and 13.3% of participants respectively and 5.1% explicitly mentioned that MC was a ritual of coming of age. There were no significant differences in participants' associations with MC between adults and young people or male and female respondents. Circumcised male respondents associated MC with improved hygiene significantly more often than uncircumcised males (23.7% vs. 10.4%, p = 0.014), but less often with initiation into manhood than uncircumcised men (0.0% vs. 9.6%, p = 0.005). Otherwise, there were no differences in general beliefs associated with MC between circumcised and uncircumcised male respondents. In Uganda, especially in the central regions, a traditional belief exists that MC protects from child sacrifice, i.e. ritual killings of male children by witch doctors. However, protection from child sacrifice was not mentioned by any of the participants.

#### Reasons to get circumcised

**Figure 2:**
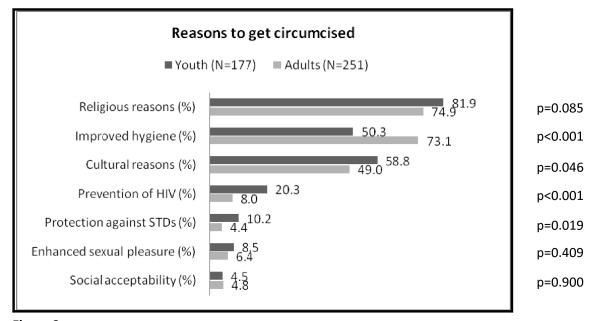
**Participants' estimates of main reasons for male circumcision (N = 428, multiple answers were possible)**.

Following the open-ended question on what participants knew about MC, we assessed participants' opinions on the main reasons for MC with multiple choice questions where more than one answer was possible. Religious reasons, followed by improved hygiene and cultural reasons were the three major reasons to become circumcised for adults and young people. However, adults mentioned improved hygiene as a reason for MC significantly more often than youths and thought that cultural reasons were less important (Figure 2). Females of any age considered religion as a more important reason for MC than males (82.5% vs. 74.1%; p = 0.036) as well as cultural reasons (57.7% vs. 49.4%; p = 0.088). Prevention of HIV was considered a reason to circumcise by 13.1% of all participants, almost twice as often as protection against other STDs. However, young people mentioned the protective effect of MC regarding HIV and other STDs significantly more often than adults. Enhanced sexual pleasure and social reasons were also mentioned, but with no differences between adults and youth (Figure 2). Male participants considered enhanced sexual pleasure twice as often a reason to circumcise than females (4.8% vs. 9.2%, p = 0.078). Circumcised male respondents did not differ from uncircumcised men in their views on the main reasons for MC, except for one: Enhanced sexual pleasure was considered a reason to get circumcised significantly more often by uncircumcised than by circumcised men (18.9% vs. 2.4%, p = 0.005).

#### Reasons not to get circumcised

Two thirds of the uncircumcised male participants reported reasons for not being circumcised (N = 93): cultural reasons (adults 30.4% vs. youths 12.8%; p = 0.038), fear of complications (23.9% vs. 29.8%; p = 0.523) and religious reasons (19.6% vs. 40.4%; p = 0.028). One fifth of adults (19.6%) said that they were not interested in the subject, although almost all of them had heard that MC reduced the risk of contracting HIV (6.4% of youths; p = 0.058). An opposition of the partner (adults) or parents (young people) was mentioned in 6.5 vs. 4.3% of the cases (p = 0.628). Accessibility of services and cost were of concern to the youth (10.6%, p = 0.023, and 6.4%, p = 0.082, respectively), but not mentioned by adults.

## Discussion

### Principal findings

This survey in rural Uganda showed a high level of awareness of MC as a protective measure against HIV among both adults and youth. In adults, the awareness of MC for HIV prevention was strongly associated with increasing educational level, younger age and male gender. In youths, higher education was not significantly associated with awareness after adjusting for potential confounders. Religious affiliation, tribal identity, employment status and circumcision status (in a subgroup analysis of men only) were not associated with awareness of MC and HIV prevention. The qualitative assessment of general knowledge and beliefs about MC showed that a lower percentage of participants associated MC with HIV prevention compared to participants' answers when prompted directly. Apart from the association with HIV prevention, participants of this survey associated MC with Muslim religion, culture, improved hygiene and the protection against STDs. Although participants were aware of the preventive effect of MC, religion, improved hygiene and culture were the main reasons given for getting circumcised by youth, adults, female and circumcised and uncircumcised male respondents. Similarly, though representing a smaller number of (male only) participants, reasons against MC were mainly cultural and religious, alongside with fear of complications of the procedure. One out of five adult men was not interested in getting circumcised at all.

### Possible limitations of the study

The data on MC were collected as part of a larger survey that aimed at developing age-specific preventive strategies against HIV for young people and adults. Districts, counties and sub-counties were selectively chosen to avoid overlap with HIV prevention programmes of other service providers. Therefore, our study population cannot be considered being representative of other rural populations in Uganda.

No clinical examinations were performed, but circumcision status was based on self-report. Therefore, we cannot rule out some misclassification of MC status as suggested in previous studies[16,18].

Assessing awareness of study participants that MC reduces the risk of contracting HIV, we did not distinguish between an awareness informed by scientific evidence as opposed to traditional beliefs in the communities. Beliefs that MC protects against sexually transmitted diseases like HIV have always existed in most of Sub-Saharan Africa [10], but are less common among traditionally non-circumcising groups such as the majority of Ugandans. In addition, awareness of MC as an HIV prevention method does not necessarily translate into high levels of acceptability or demand of MC services as this benefit might be only one aspect leading to the decision to become circumcised. In a Kenyan study on predictors of circumcision preference, beliefs about the protective effect of MC against HIV were not significantly associated with willingness to become circumcised after adjusting for other variables[19] However, a study from South Africa showed that men who were not aware of any health-related aspects of MC were the least willing to be circumcised[20].

As only one of the youth participants had never gone to school and very few had completed tertiary or university education, we defined the reference category for the variable educational level as "no school/primary school" and combined secondary and higher education. However, this may not explain why the better educated youths knew less about the prevention of HIV through MC compared to those with a lower educational level.

We note as a further limitation that for some of the categories, e.g. casual/manual work, it was not possible to obtain risk estimates because of the low numbers in these categories. However, collapsing these categories with other categories would not have improved the interpretation of this variable.

Due to our stratified analyses following the a priori separation between youths and adults in the design of the overall survey, effect estimates of the two samples cannot be directly compared.

### Meaning of the study and implications

While the greatest impact of MC on the prevalence of HIV over the following 10 years can be achieved by targeting men at highest risk of HIV exposure between 25-34 years, the greatest population level benefit in the long-term would be achieved by targeting adolescents and young adults before sexual debut[21,22] However, awareness and attitudes towards MC have not been studied specifically among the youths. In our study, age only marginally influenced awareness of MC as partly protective against HIV. This is in line with qualitative research on acceptability of MC from South Africa indicating that ideas about MC are not limited to certain age groups[13] However, age is likely to influence men's actual decision to become circumcised - or to rather support circumcision for their male offspring[11,12,23].

Reported circumcision rates of male participants (37.3%) were above national average, which can be explained by the relatively high percentage of Muslims, living in Mpigi (and reporting to be circumcised in 96.7% of the cases) and ethnic mixing with traditionally circumcising groups in Kayunga. Being circumcised was not associated with increased awareness of the protective effect of MC against HIV.

Among adults, higher educational level was an important determinant of awareness of MC as a means of HIV prevention. This trend has also been observed in a study on acceptability of MC in Malawi, where educated participants were more likely to be knowledgeable about MC and HIV[24] Circumcision for health reasons was more common among traditionally non-circumcising participants with secondary education in a Tanzanian study, but this was rather attributed to contact with students from different ethnic backgrounds through secondary schools than secondary education itself[16] Changes in existing practices, like the medicalization of MC in Nigeria have also been said to depend on education[25] In Uganda, where only 45% of men and 31% of women have completed primary education or more[8], our findings emphasize the need to target those who have not had access to formal education through informational campaigns on medical MC for HIV prevention. In our study, the media were reported to be the main source of information on MC and its protective effect against HIV. However, exposure to the media is also dependent on education[8].

We observed a trend for increased awareness of MC for HIV prevention among adult male participants. In the light of findings from other studies however, it is clear that communities do not treat MC as an exclusively male issue, but that mothers and wives/partners participate in the decision about MC[13,15] Among traditionally circumcising groups in Uganda, MC is usually not an optional procedure, but the timing of MC can be an individual decision (whether or not the adolescent or young man feels ready to undergo MC) or a family decision (e.g. certain family customs exist whereby sons are always circumcised at a certain age). Male and female participants of a recent study on acceptability of MC among non-circumcising communities in Uganda thought that parents should decide together whether or not to circumcise their sons, or leave the decision to their sons provided they were old enough. Others felt, it should be solely the father or the mother, if the father was not available. Acceptability of circumcision for their sons was higher in women than in uncircumcised men after exposure to a brief health message on MC for HIV prevention[15] Similarly, especially female participants of a Kenyan study with a higher educational level were more likely to prefer their partners to be circumcised[19] Informational campaigns creating awareness of MC as a means of reducing the risk of HIV infection should thus be targeted to men and women in order to reach everyone involved in the decision making on MC.

Prevention of HIV, religion, culture, hygiene and protection against HIV were the most common factors that were associated with MC by participants of our study. Although HIV prevention was most often mentioned when participants were asked what they had "ever heard" of MC, it was not mentioned as a reason to become circumcised to the same extent. A (qualitative) study from Zambia showed similar results: The majority of males and females believed that MC protected against STDs, including HIV, but felt they needed clear endorsement of MC by the government and community leaders before acting on their beliefs and getting circumcised or sending their sons[26].

For adults, we collected information also on their social function/role and grouped them into parents, teachers, and Sengas/Kojjas. These three groups represent relevant communicators to children and young people in the communities. The need for an appropriate source of sexual health information for adolescents in rural communities is not met by the modern health care system alone. The Senga institution, as the traditional channel for socializing adolescents into sexual matters among many ethnic groups in Uganda, could provide an opportunity to introduce information on MC as part of other measures for HIV prevention as shown for other sexual and reproductive health matters elsewhere in Uganda[27] We did not find any difference in basic awareness of MC and HIV between parents, teachers and Sengas/Kojjas of our study. However, given the small sample size, it is difficult to appreciate the representativeness of these findings. Further studies are warranted to explore the acceptability and feasibility of approaching traditional counsellors as communicators on MC for HIV prevention.

## Conclusions

In this Ugandan survey, youth, women, and participants with a low educational level were considerably less informed about MC as a preventive measure to reduce the risk of contracting HIV. Further studies are warranted to confirm the present findings in a representative sample applicable to rural Uganda as a whole. Young people should continue to be in the focus of programmes aiming at a scale-up of demand for medical MC services. Women, playing an important role in the decision-making for and against MC for their partners and sons should be increasingly targeted by information campaigns about positive health effects of medical MC. Although but one aspect influencing the decision whether or not to become circumcised, increased awareness of the benefits of MC for HIV prevention is an important step towards informed decision-making among the general population. Community-based approaches to reach out to those who do not have access to education and/or the media as source of information should be further explored.

## Competing interests

The authors declare that they have no competing interests.

## Authors' contributions

AW participated in the development of questionnaires for both the overall project and the present study, designed the present study, performed the statistical analyses and wrote the manuscript. FN participated in the development of the study design and questionnaires for the overall project, trained and supervised research assistants, was responsible for data management and critically reviewed the manuscript. RH participated in the development of the study design for the overall project, supervised data collection and management and critically reviewed the manuscript. TK supervised the development of the analysis plan, supervised and participated in the statistical analyses and in the writing of the manuscript. DB was the principal investigator of the overall project, received the funding, developed the study design and critically reviewed the manuscript. All authors have read and approved the final manuscript.

## Pre-publication history

The pre-publication history for this paper can be accessed here:

http://www.biomedcentral.com/1471-2458/10/209/prepub
